# Autophagy modulates mesenchymal-to-endothelial transition via p53

**DOI:** 10.18632/aging.104065

**Published:** 2020-11-13

**Authors:** Jie Hu, Shuting Kong, Tiancheng Dong, Zhiwei Lin, Qihao Zhang, Xingxing Chen, Yongsheng Gong, Xiaofang Fan, Mingyu He, Hao Zhou

**Affiliations:** 1 Department of Cardiology, The First Affiliated Hospital of Wenzhou Medical University, Wenzhou 325000, China; 2 Department of Hypoxia Medical Research Laboratory, Wenzhou Medical University, Wenzhou 325000, China; 3 Vascular Medicine Institute, University of Pittsburgh, Pittsburgh, PA 15260, USA

**Keywords:** mesenchymal-to-endothelial transition, autophagy, p53, endothelial cells, fibrosis

## Abstract

Mesenchymal-to-endothelial transition (MEndT) is one of the mechanisms that influences cardiac fibrosis, which is a key process in cardiac remodeling. It has been reported that autophagy inhibits endothelial cell transition. However, whether autophagy could modulate MEndT in cardiac fibrosis has not yet been investigated. Here, we discussed the association between autophagy and MEndT and its possible mechanism. In this study, we induced endothelial-to-mesenchymal transition using transforming growth factor-β to generate mesenchymal cells and fibroblasts in wild-type human umbilical vein endothelial cells and cells with p53 knockout or overexpression. Then, autophagy was induced by Earle's balanced salt solution (EBSS) and was inhibited by bafilomycin A1 or lentivirus-ATG5-shRNA. The expression levels of MEndT and the autophagy markers CD31, VE-Cadherin, Vimentin, α-SMA, LC3, p62 and p53 were examined. We found that activation of autophagy could promote MEndT and increase cytoplasmic and total expression of p53, that but nuclear p53 expression was decreased, and that inhibition of autophagy activation could reverse the effect of EBSS. Moreover, after knockout of nuclear p53, autophagy promoted MEndT, while autophagy inhibited MEndT in p53 overexpressing cells. Our results demonstrate that autophagy modulate MEndT by nuclear p53 provide a new strategy for the treatment of fibrosis diseases.

## INTRODUCTION

Cardiac fibrosis, nonphysiological wound healing in the heart characterized by the excessive accumulation of fibroblasts and extracellular matrix [1–3], can impair cardiac function and lead to the development of congestive heart failure. During the process, cardiac fibroblasts (CFs) respond to pathological stress and environmental stimulation by transforming into myofibroblasts that express elevated levels of various proinflammatory and profibrotic factors that directly contribute to inflammatory cell infiltration and fibroblast proliferation, secrete high levels of matrix metalloproteinases and other extracellular matrix (ECM)-degrading enzymes that facilitate fibroblast migration, and contribute to the deposition of collagen and other ECM proteins, leading to scar formation [4].

MEndT is a biological process by which mesenchymal cells transform into endothelial cells. Eric Ubil et al found that CFs acquired an endothelial cell phenotype through MEndT, promoted angiogenesis, improved cardiac repair, and inhibited cardiac fibrosis [5]. MEndT was also reported in juvenile angiofibromas (JAs) [6] and Kaposi sarcoma (KS) [7]. Recent studies have shown that endothelial progenitor cell (EPC)-derived exosomes inhibited cardiac fibrosis by promoting MEndT and angiogenesis via high mobility group box 1 protein B1 (HMGB1) and Y box binding protein 1 (YBX-1) [8, 9]. All lines of evidence show that MEndT in cardiac remodeling may provide a potential target for the treatment of fibrotic diseases.

Autophagy is a highly conserved intracellular phenomenon that occurs in eukaryotic cells. It plays a role in self-protection by promoting the recycling of cytoplasmic components, such as proteins and organelles [10–12] and has also been linked with programmed cell death. Recent studies have shown that autophagy is involved in the formation of fibrosis diseases in the heart, kidney and other organs [13]. Nonphysiological increased or decreased levels of autophagy can promote cardiac interstitial fibrosis, causing heart failure [14, 15]. Therefore, autophagy may play an important role in fibrosis diseases.

p53 has been widely studied as a tumor suppressor in humans and other mammals [16] and has a dual role in fibrosis. p53 inhibits collagen synthesis [17], but accumulation of p53 increases endothelial cell death and rarefaction of cardiac microvasculature to promote the development of fibrosis [18]. p53 also plays a dual role in the regulation of autophagy depending on the different localization in cells. Nuclear p53 promotes autophagy activation by either relying on or not relying on transcription. In the cytoplasm, p53 inhibits autophagy, which may involve the regulation of glycolysis [19].

Relevant studies have shown that autophagy could inhibit endothelial-to-mesenchymal transition (EndMT) [20–22], a process opposite to MEndT and one that contributes to the differentiation of cardiac fibroblasts (CFs) that induce cardiac fibrosis [23, 24]. Moreover, the expression of total p53 was increased in MEndT [5]. Therefore, we asked whether autophagy could modulate MEndT through p53. To test this hypothesis, in the present study, we investigated the role of autophagy in MEndT using human umbilical vein endothelial cells (HUVECs) experiments. The results demonstrated that autophagy contributes to MEndT by regulating the expression of nuclear p53.

## RESULTS

### Autophagy induced by EBSS modulates MEndT

**Figure 1:**
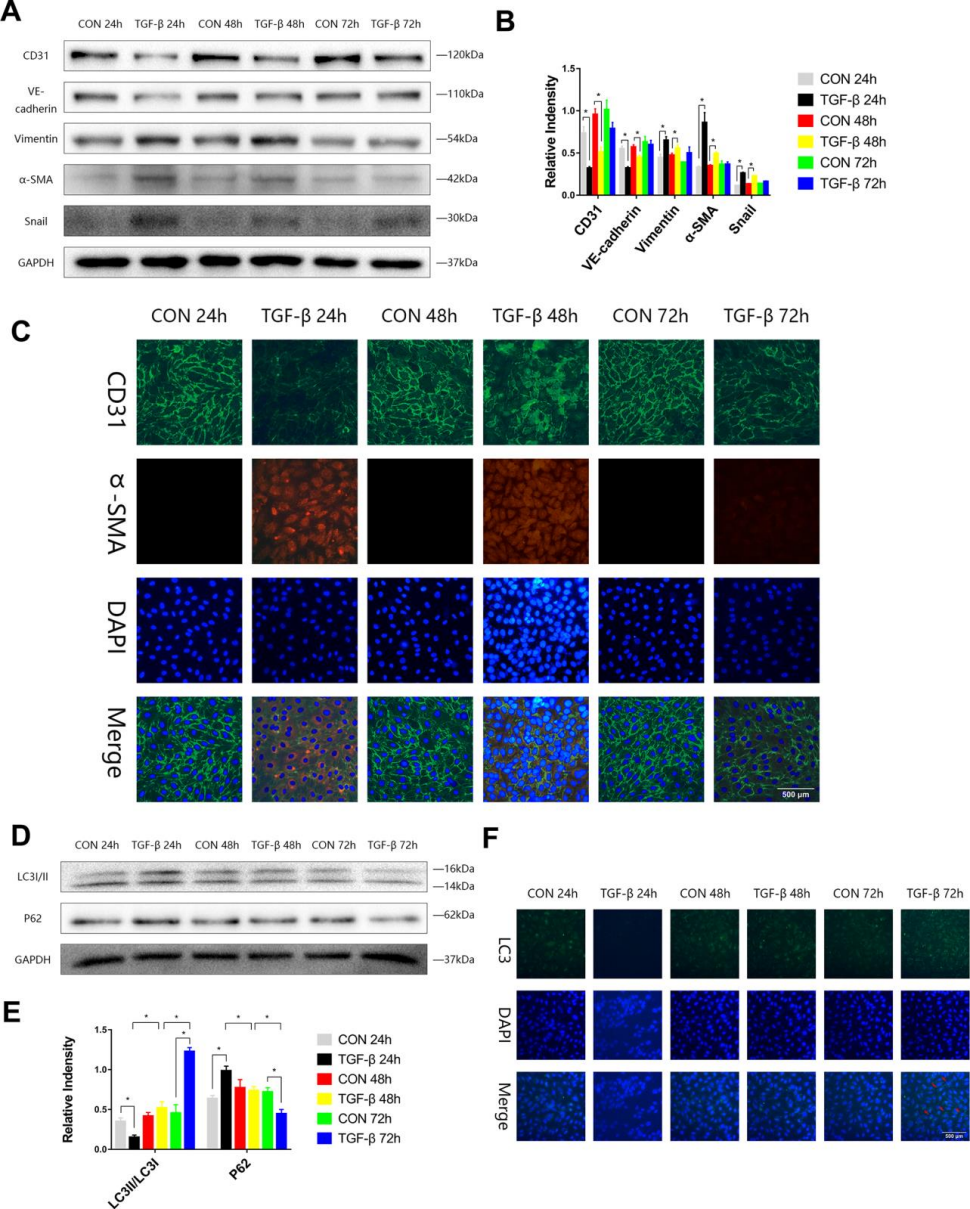
**The autophagy level was changed during TGF-β-induced EndMT.** (**A**, **B**) HUVECs were exposed to TGF-β (20 ng/ml) for 24, 48 and 72 h. Endothelial and mesenchymal markers were assessed using antibodies against CD31, VE-cadherin, Vimentin, α-SMA and Snail via Western blot. (**C**) Representative images (Scale bars= 500 μm) of immunofluorescence after staining of CD31 and α-SMA 24, 48 and 72 h after TGF-β stimulation. (**D**, **E**) Immunoblots were probed for autophagy markers LC3-II/LC3-I and p62 of cell lysates harvested from HUVECs treated with TGF-β for 24, 48 and 72 h. (**F**) Representative fluorescence microphotographs (Scale bars= 500 μm) of HUVECs immunostained for LC3. Bar graphs represent data that were from three independent experiments, and data represent the means±SEM. Unpaired T test (*P<0.05) was used to compare the significances between two groups.

To stimulate the transformation of endothelial cells into mesenchymal cells during cardiac fibrosis and to obtain a special type of cells that expressed both endothelial and mesenchymal markers, we induced EndMT by transforming growth factor-β (TGF-β) in HUVECs, and the expression levels of endothelial (CD31, VE-Cadherin), mesenchymal markers (Vimentin, α-SMA), and transcription factor (Snail) in HUVECs were assayed by immunoblotting in the EndMT process. We observed increases in Vimentin, α-SMA, and Snail expression and decreases in CD31 and VE-Cadherin expression at 24 or 48 h (Figure 1A, 1B). Immunofluorescence staining also showed the same trend consistent with the Western blot results (Figure 1C). During the process of EndMT, the expression of p62 was higher, and LC3-II/LC3-I expression was lower at 24 h. The expression of p62 was then downregulated, and LC3-II/LC3-I expression was enhanced at 72 h (Figure 1D, 1E). Immunofluorescence staining of LC3 also showed that during EndMT induced by TGF-β, autophagy was subject to variation (Figure 1F).

**Figure 2:**
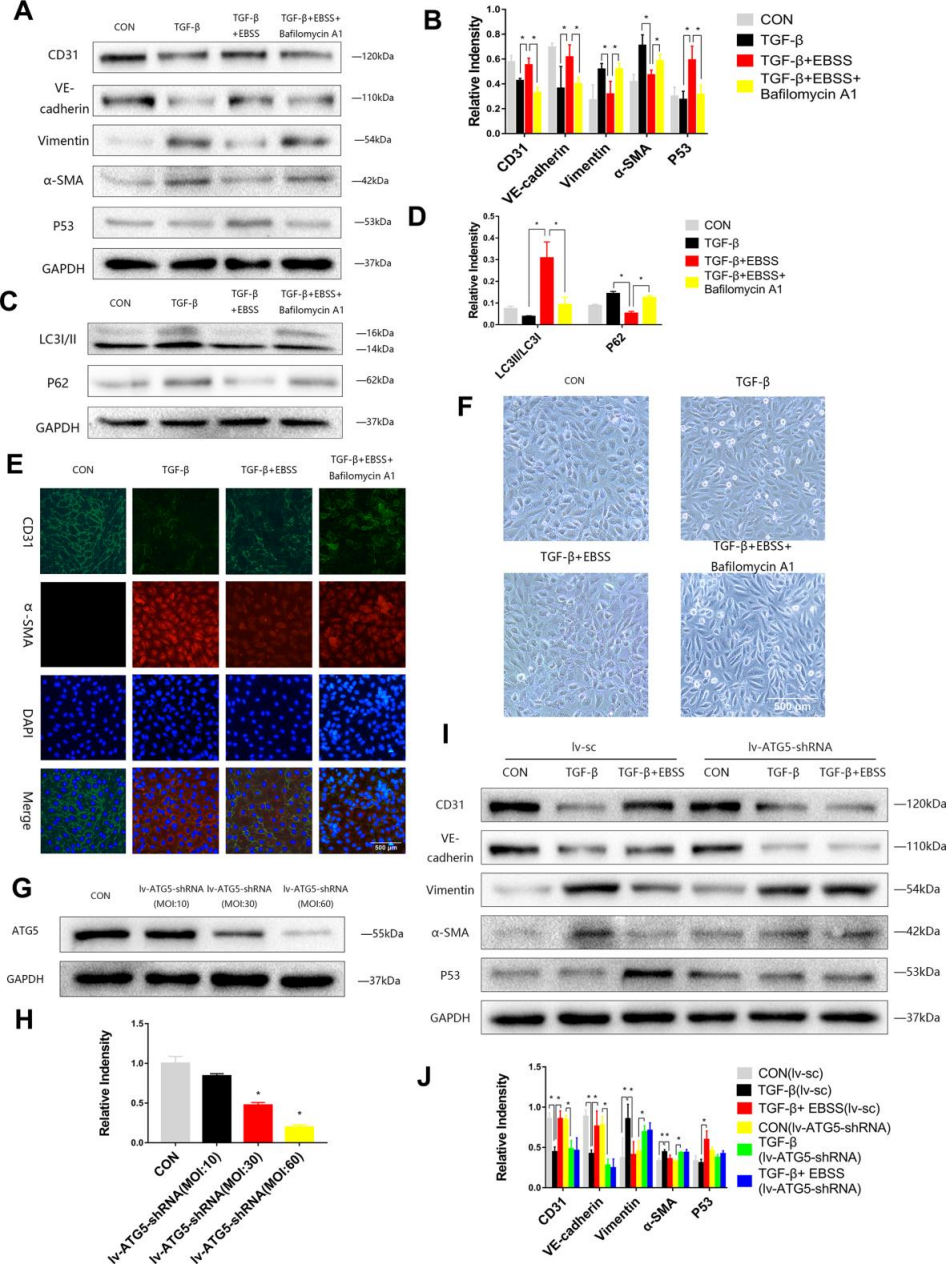
**Autophagy modulated MEndT.** (**A**–**D**) Cells were subjected to TGF-β (20 ng/ml) for 24 h and then treated with EBSS for 4 h, with pretreatment with bafilomycin A1 for 2 h or not. CD31, VE-cadherin, Vimentin, α-SMA, LC3-II/LC3-I, p62 and p53 levels were analyzed by immunoblot. (**E**) MEndT was assessed by representative images (Scale bars= 500 μm) of immunofluorescence after staining of CD31 and α-SMA. (**F**) Representative microphotographs (Scale bars= 500 μm) of HUVEC morphological changes. (**G**, **H**) Cells were infected with GFP-labeled lv-ATG5 for 24 h, and infection was assessed by the expression of ATG5. (**I**, **J**) Cells were treated with TGF-β and EBSS as described above. CD31, VE-cadherin, Vimentin, α-SMA and p53 levels were analyzed by immunoblot. Bar graphs represent data from three independent experiments, and data represent the means±SEM. Unpaired T test (*P<0.05) was used to compare the significances between two groups.

**Figure 3:**
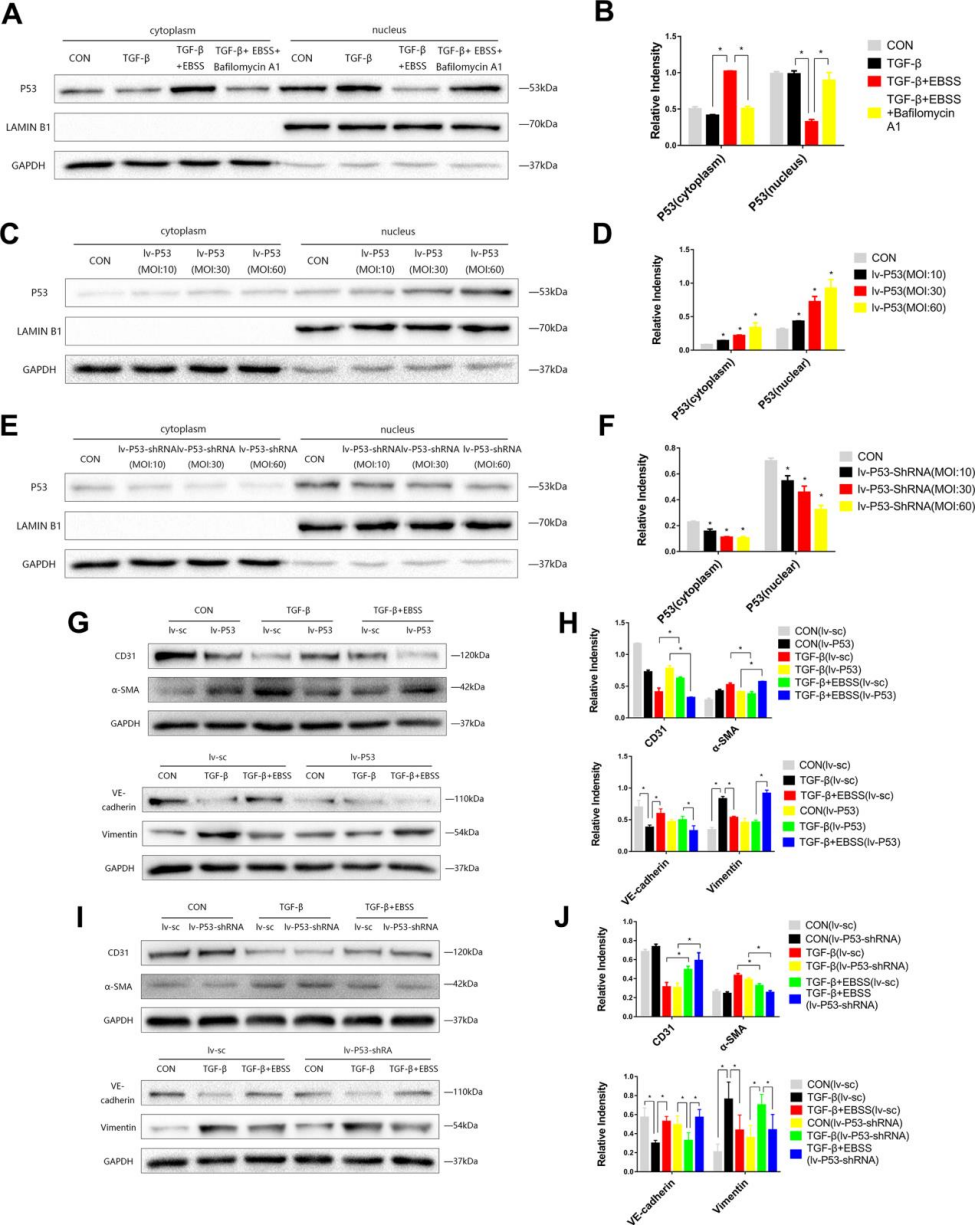
**MEndT was modulated by autophagy by regulating the expression of nuclear p53.** Cells were treated with TGF-β and EBSS as described above. (**A**, **B**) p53 expression in the cytoplasm and nucleus was evaluated by Western blot. (**C**, **D**) Cells were infected with lv-p53 for 24 h, and infection was assessed by the expression of p53. (**E**, **F**) p53 expression in cells treated with lv-p53-shRNA. (**G**, **H**) After overexpressing p53, cells were treated by the method described above, CD31, VE-cadherin, Vimentin and α-SMA levels were analyzed by immunoblot. (**I**, **J**) CD31, VE-cadherin, Vimentin and α-SMA expression in cells treated with lv-p53-shRNA using the method described above. Bar graphs represent data from three independent experiments and data represent the means±SEM. Unpaired T test (*P<0.05) was used to compare the significances between two groups.

To investigate the interaction between autophagy and MEndT, HUVECs were treated with TGF-β for 24 h and then subjected to EBSS for 4 h. Lower Vimentin and α-SMA expression and higher CD31 and VE-Cadherin expression were observed after the EBSS induced autophagy compared to the cells only treated with TGF-β. If the autophagy was inhibited by being treated with Bafilomycin A1, an autophagy inhibitor, the expression levels of CD31, VE-Cadherin, Vimentin and α-SMA could not be significantly altered after EBSS treatment (Figure 2A–2D). Immunofluorescence staining also showed that EBSS increased the expression of CD31 and decreased the expression of α-SMA, while Bafilomycin A1 could reverse the effect of EBSS (Figure 2C). In Figure 3F, TGF-β-treated HUVECs showed an altered morphology, resembling fibroblasts. Additionally, EBSS-induced autophagy converted the cell morphology similar to the morphology of EC control. The MEndT morphology change was not observed when the autophagy was inhibited by Bafilomycin A1.

To further investigate the function of autophagy, cells were infected with Lv-ATG5-shRNA or a negative control lentivirus for 24 h, following replacement with a normal culture medium for 24 h. Then, we visualized the GFP-labeled marked the lentivirus and performed Western blot analysis to confirm knockdown of ATG5 (Figure 2G, 2H). After the cells were stably transfected, the cells were treated with TGF-β and EBSS as described above, and we found that the lentivirus itself did not influence the effect of EBSS on MEndT, while after knockdown of ATG5, EBSS could not decrease the expression of CD31 and VE-Cadherin and increased Vimentin and α-SMA expression (Figure 2I, 2J).

### Autophagy modulates MEndT by regulating the expression of nuclear p53

According to previous research, we hypothesized that autophagy could modulate MEndT via p53 [5]. EBSS-induced autophagy could increase the expression of total p53, while autophagy inhibition reduced the expression of total p53 (Figure 2A, 2B). As p53 generally acts as a nuclear transcription factor, we found that the expression of cytoplasmic p53 was increased during autophagy-modulated MEndT, while nuclear p53 expression was decreased, and the autophagy inhibitor Bafilomycin A1 could reverse the effect of EBSS (Figure 3A, 3B).

To further investigate the correlation of p53 and MEndT, cells were infected with Lv-p53, Lv-p53-shRNA or their negative control lentivirus for 24 h, following replacement with normal culture medium for 24 h. GFP marked the lentivirus, and Western blot analysis was used to test the effect of p53 overexpression (Figure 3C, 3D) or p53 knockdown (Figure 3E, 3F). After the transfected cell lines were stable, cells were treated with TGF-β followed by EBSS treatment as described above. We found that lentivirus transfection per se did not affect the EBSS-promoted MEndT and that EBSS was less efficient in facilitating MEndT with p53 overexpression, as demonstrated by the lower expression levels of CD31 and VE-Cadherin and increased Vimentin and α-SMA expression (Figure 3G, 3H). In addition, p53 knockout by Lv-p53-shRNA transfection had an opposite effect, i.e., EBSS induced a more EC-like phenotype (Figure 3I, 3J). These data suggest that by regulating the expression of nuclear p53, which generally acts as a nuclear transcription factor, autophagy differentially contributes to MEndT.

## DISCUSSION

In this study, we found that autophagy regulates MEndT by altering the expression level of p53 in the nucleus. HUVECs were treated with TGF-β 24 h and then subjected to EBSS to induce autophagy. Variations in CD31, VE-Cadherin, Vimentin and α-SMA expression suggested the existence of MEndT, while Bafilomycin A1 and lv-ATG5-shRNA could eliminate the role of EBSS in this process. Therefore, we hypothesized that autophagy plays an important role in MEndT. Then, we used lentivirus transfection to build cell models with overexpression and knockdown of p53 expression and found that autophagy could modulate MEndT by regulating nuclear p53. To the best of our knowledge, this is the first study of the relationship between autophagy and MEndT, which could provide new insights for the treatment of fibrosis.

MEndT refers to the process in which mesenchymal cells lose the relevant phenotype and then obtain the phenotype of endothelial cells [5]. During this process, endothelial markers such as CD31 and VE-cadherin are upregulated, while the expression levels of vimentin and α-SMA are decreased. Eric Ubil et al. first observed this phenomenon in a model of cardiac ischemia-reperfusion injury and found that p53 is involved in the regulation of this process [5]. Recently, some scholars found that CF-induced MEndT was less angiogenic than preexisting endothelial cells in injured hearts, but they still regarded MEndT as a cellular and molecular mechanism of cardiac angiogenesis that could alleviate cardiac fibrosis [25, 26], indicating that MEndT may be a new target for the treatment of fibrotic diseases.

To observe the phenomenon of MEndT, We induced EndMT by TGF-β in HUVECs to obtain a special type of cells that express both endothelial and mesenchymal markers and then used EBSS treatment to induce autophagy. The results showed that compared with the TGF-β group, the cells treated by EBSS changed from spindle to round shape, and the expression levels of CD31 and VE-Cadherin increased, while the expression levels of Vimentin and α-SMA decreased. These results preliminarily suggest that MEndT is a biological process in which mesenchymal cells lose their mesenchymal phenotype and acquire the endothelial cell phenotype, and some of the results were consistent with our previous research [27].

During autophagy, the expression level of p62 was downregulated [28, 29], accompanied by the transformation from LC3 I to LC3 II [30]. In the process of EndMT in HUVECs induced by TGF-β, we found that the autophagy expression level was changed, consistent with the findings of Wang J [21]. Thus, based on the above cell model, the autophagy inhibitors bafilomycin A1 and lv-ATG5-shRNA were used to inhibit autophagy activation, and both bafilomycin A1 and lv-ATG5-shRNA could abolish the effect of EBSS. The results suggest that autophagy has a regulatory effect on MEndT.

MEndT regulated by p53 expression has been reported in previous studies [5]. In our study, we also found that EBSS could increase the total p53 expression of the ECs treated with TGF-β, and this increase could also be inhibited by bafilomycin A1 and lv-ATG5-shRNA. As a nuclear transcription factor, p53 performs functions in the nucleus. Therefore, we tested p53 expression in the cytoplasm and nucleus separately; cytoplasmic p53 expression was increased and nuclear p53 expression was decreased in MEndT modulated by autophagy, and Bafilomycin A1 and lv-ATG5-shRNA could reverse the transition. We transfected p53 knockdown and overexpressing cell models to further confirm the role of p53 in the MEndT modulated by autophagy. We found that autophagy could promote MEndT in the nuclear p53 low expression model, while autophagy could inhibit MEndT of the nuclear p53-overexpressing cells. Thus, we determined that autophagy modulates MEndT and may be regulated by nuclear p53.

The study only discussed whether autophagy is correlated with MEndT and preliminarily discussed the role of p53 in autophagy regulation of MEndT and its mechanism. The detailed mechanism remains to be further explored, including (1) how autophagy regulates MEndT through nuclear p53; and (2) which cell type should be mainly targeted by autophagy intervention. In the process of inducing EndMT, endothelial cells may differentiate into mesenchymal cells and fibroblasts; however, there are currently technical limitations on how to separate three different cell types.

Overall, we demonstrated that autophagy contributes to MEndT via nuclear p53 and that autophagy seems to be an attractive therapeutic target to modulate the disease progression of fibrotic diseases.

## MATERIALS AND METHODS

### Cell culture and treatment

HUVECs were purchased from Shanghai Cell Bank (Shanghai, China) and cultured in DMEM (#C11995500BT, Gibco, New York, USA) with 1% penicillin-streptomycin (P/S, #P1400, Solarbio, Beijing, China) and 10% heat-inactivated fetal bovine serum (FBS, #16000-044, Gibco) in humidified incubators at 37°C and 5% CO_2_. Cells were transferred to 6-well plates at a concentration of 5×105 cells per well and divided into four groups: (1) cells cultured under normal conditions as a control; (2) cells treated with TGF-β [31, 32] (20 ng/ml, #100-21, PeproTech, New Jersey, USA); (3) cells treated with TGF-β for 24 h first, and then subjected to EBSS (#H2045, Solarbio, Beijing, China) for 4 h; and (4) cells treated with TGF-β (20 ng/ml) for 24 h, and then subjected to EBSS and bafilomycin A1 [33] (100 nM, #A8510, Solarbio, Beijing, China) for 4 h.

### Antibodies and materials

Anti-p53 (#2524S) and anti-Snail (#C15D3) were purchased from Cell Signaling Technology (Boston, USA), anti-CD31 (#ab28364), anti-VE-cadherin (#ab33168), anti-LC3 (#ab48394), anti-p62 (#ab56416), and anti-ATG5 (#ab108327) were obtained from Abcam (Cambridge, UK), anti-α-SMA (#A5228) was purchased from Sigma (St. Louis, USA), and anti-Vimentin (#10366-1-AP) was obtained from Proteintech (Wuhan, China).

### Western blot analysis

Cells were lysed with RIPA (#P0013B, Beyotime, Shanghai, China) buffer with PMSF (#ST506, Beyotime, Shanghai, China) or using a protein extraction kit (#R0050, Solarbio, Beijing, China) to isolate proteins in the nucleus and cytoplasm. The protein concentration was quantified using a BCA Protein Quantification Kit (#20201ES76, YEASEN, Shanghai, China). Equivalent protein amounts were separated by SDS-PAGE and transferred to PVDF membranes (#ISEQ00010, Millipore, Massachusetts, USA). The membranes were blocked with 5% nonfat powdered milk in TBST, followed by incubation with primary antibodies overnight at 4°C and then appropriate secondary antibodies for 1 h at room temperature. The protein bands were detected with SuperSignal West Pico PLUS Chemiluminescent Substrate (#34580, ThermoFisher, Massachusetts, USA).

### Immunofluorescence staining

Immunofluorescence staining was used to assess the MEndT of HUVECs. Briefly, cells were incubated with primary antibodies, including anti-CD31 (1:20), anti-α-SMA (1:500) and anti-LC3 (1:200), in 5% bovine serum albumin at 4°C overnight and then were washed and incubated with a mixture of secondary antibodies conjugated to Alexa Fluor 594 (#33212ES60, YEASEN, Shanghai, China) or Alexa Fluor 488 (#33106ES60, YEASEN, Shanghai, China) for 1 h at 37°C. The cells were then washed with PBS several times and incubated with DAPI for 5 min to counterstain the nucleus. All images were captured with a fluorescence microscope.

### Lentivirus constructions and infection

Lentivirus-ATG5-shRNA (lv-ATG5-shRNA), lentivirus-p53 (lv-p53) and lentivirus-p53-shRNA (lv-p53-shRNA) were obtained from Hanbio. The ATG5 shRNA target sequence is 5′- GCAGATGGACAGTTGCACACA -3′, and a scramble version was used as a control, 5′- GTTCTCCGAACGTGTCACGT -3′. The p53 shRNA target sequence is 5′- GAGTGGAAGGAAATTTGCGTGTGGAGAGTGGAAGGAAATTTGCGTGTGGA -3′, and a scramble version was used as a control, 5′- TTCTCCAACGTGTCACGTAA -3′. Cells were transfected for 48 h with vectors expressing ATG5-shRNA, p53, p53-shRNA or their control sequence using lentivirus according to the instructions provided by the manufacturer.

### Statistical analysis

Data represent the means±SEM. A p value < 0.05 was considered statistically significant. Analyzes were performed using GraphPad 6 and SPSS statistical software, version 18.
